# Clinical outcome of an SCNT-derived *MSTN* knockout buffalo: a case study

**DOI:** 10.3389/fgeed.2026.1822169

**Published:** 2026-06-09

**Authors:** Priyanka Singh, Kartikey Patel, Gaurav Tripathi, Shavi Verma, Kusum Kashyap, Ranjeet Verma, Babu Lal Jangir, Manoj Kumar Singh, Naresh L. Selokar

**Affiliations:** 1 Animal Biotechnology Division (ABTD), ICAR-National Dairy Research Institute (NDRI), Karnal, Haryana, India; 2 Department of Veterinary Pathology, College of Veterinary Sciences, Lala Lajpat Rai University of Veterinary and Animal Sciences (LUVAS), Hisar, India

**Keywords:** buffalo, genome editing, immunometabolic regulation, muscle growth, myostatin knockout, somatic cell nuclear transfer

## Abstract

Myostatin (*MSTN*) gene knockout has attracted considerable interest for enhancing meat production in livestock species due to its well-established role as a negative regulator of skeletal muscle growth. In this study, we report the clinical outcome of a bi-allelic *MSTN* knockout buffalo produced via somatic cell nuclear transfer (SCNT) that failed to survive beyond 100 days of age. The *MSTN* knockout calf exhibited a double-muscling phenotype, which was validated by changes in mRNA expression of transcripts associated with increased muscle mass and reduced subcutaneous fat. In addition, the calf had a telomere length similar to that of age-matched calves produced through artificial insemination. However, serum biochemistry analysis revealed decreased levels of globulin, GGTP, ALP, and bilirubin, alongside increased concentrations of AST, urea, blood urea nitrogen, uric acid, phosphorus, and potassium. Cytokine profiling (including IFN-γ, IL-1α, IL-1β, IL-4, IL-6, IL-8, IL-10, IL-17α, IL-36Rα, TNF-α, MIP-1α, MIP-1β, MCP-1, IP-10, and VEGF-α) demonstrated dysregulation of several inflammatory and chemotactic mediators. This case report highlights potential health and welfare challenges associated with producing *MSTN* knockout buffalo through cloning-based approaches.

## 1 Introduction

Genome editing technologies, particularly the CRISPR/Cas9 system, have revolutionized livestock biotechnology by enabling precise and heritable modifications at specific locations in the genome (Tait-Burkard et al., 2018). These tools have been used to introduce valuable traits in livestock species, such as faster growth, better milk quality, increased disease resistance, and improved reproduction (Ledesma and Van Eenennaam, 2024; Yuan et al., 2024). The myostatin (*MSTN*) gene is the most widely studied target in this field due to its well-established role as a negative regulator of skeletal muscle growth (McPherron and Lee, 1997). When the *MSTN* gene is disrupted (loss-of-function mutations), it leads to double-muscling phenotypes (McPherron and Lee, 1997). This is characterized by an increase in both the size and the number of muscle fibers in livestock species, such as goats (Ni et al., 2014), sheep (Li et al., 2016), pigs (Zhu et al., 2020), cattle (Gim et al., 2022), and buffalo (Selokar et al., 2025). However, despite the benefits for meat production, there are concerns regarding the physiological consequences of disrupting the *MSTN* gene because this gene also helps regulate other vital biological processes, and its removal may have a broad range of impacts on an animal’s physiological development (Xin et al., 2020; Tang et al., 2021; Wen et al., 2022; Gu et al., 2023; Zhao et al., 2025a; b).

Recently, we reported the successful birth of an *MSTN* gene knockout buffalo generated using CRISPR/Cas9-mediated genome editing followed by somatic cell nuclear transfer (SCNT) (Selokar et al., 2025). Although the edited calf exhibited markedly accelerated growth compared with age-matched counterparts, it died at 100 days of age. Here, we present the clinical outcome of this calf by analyzing muscle transcript profiles, serum biochemical parameters, circulating cytokine signatures, and telomere length. Despite the limited sample size and the potential influence of SCNT-associated effects, the findings from this case report provide important preliminary insights to guide future efforts in generating *MSTN*-knockout buffalo via SCNT methods. These observations emphasize the need to optimize genome-editing and cloning strategies while prioritizing animal health, physiological stability, and long-term viability.

## 2 Materials and methods

### 2.1 Animal management and sample collection

The experiments were approved by the Institutional Biosafety Committee at ICAR-National Dairy Research Institute, Karnal (IBSC/01/2021). All procedures followed the guidelines from the Department of Animal Husbandry, India (V-11011(13)/19/2021-CPCSEA-DADF). The knockout calf in this study was previously produced using CRISPR/Cas9-mediated genome editing followed by SCNT (Selokar et al., 2025). The *MSTN* knockout calf and wild-type buffalo calves, which were produced through artificial insemination, were raised under identical management conditions.

### 2.2 Gene expression analysis

Total RNA was extracted from gluteal muscle biopsies collected at day 60 post birth from the *MSTN* knockout calf and three age-matched controls using TRIzol reagent (Invitrogen, United States), followed by DNase I treatment to remove genomic DNA contamination. The RNA concentration and purity were determined spectrophotometrically at 260 nm. First-strand cDNA was synthesized from 1 µg of total RNA using the SuperScript® III First-Strand Synthesis Kit (Invitrogen, Carlsbad, CA, United States) according to the manufacturer’s protocol. Quantitative real-time PCR (qRT-PCR) was performed on a CFX96 Real-Time PCR System (Bio-Rad, United States) using Maxima® SYBR Green Master Mix (Invitrogen, United States) and gene-specific primers (Supplementary Table S1). The cycling conditions were 95 °C for 2 min, followed by 40 cycles of 95 °C for 45 s, 60 °C for 30 s, and 72 °C for 30 sec. The specificity of amplification was confirmed by melt-curve analysis and 2% agarose gel electrophoresis. The relative gene expression levels were calculated using the 2^−ΔΔCt^ method (Livak and Schmittgen, 2001), with GAPDH serving as the internal control. Each reaction was performed in triplicate, and three independent experiments were conducted.

### 2.3 Serum biochemical analysis

As part of standard management practices, 10 mL of blood was collected from the newborn calves, including the *MSTN* knockout calf, within 24 h of birth, following colostrum feeding, using serum activator tubes. Samples were allowed to clot at room temperature for 4 h and then centrifuged at 1,000 × g for 15 min (18 °C–24 °C). The separated serum was aliquoted and stored at −80 °C until analysis. Serum biochemical parameters, including AST, ALT, GGTP, alkaline phosphatase, total bilirubin, total protein, albumin, globulin, albumin-to-globulin ratio (A:G), urea, BUN, creatinine, uric acid, calcium, phosphorus, sodium, potassium, and chloride, were measured at a certified veterinary diagnostic laboratory (Dr. Lal PathVet, New Delhi, India; https://www.pathvets.in) following standard protocols. Ten age-matched control buffalo calves were used for comparative observations.

The cytokine levels were quantified using a bovine cytokine magnetic bead multiplex assay (BCYT1-33 K-PX15, Merck Millipore, Germany) according to the manufacturer’s instructions. Data were acquired using a Bio-Plex MAGPIX reader and analyzed with Bio-Plex Manager 6.0 software. The panel included pro-inflammatory cytokines (IFN-γ, IL-1α, IL-1β, IL-6, IL-8, IL-10, IL-17α, TNF-α, MIP-1α, MIP-1β, and MCP-1), anti-inflammatory cytokines (IL-4 and IL-36Rα), and chemokines/growth factors (IP-10 and VEGF-α). Three pooled samples, each comprising serum from three age-matched control buffalo calves, were analyzed, with each sample run in three technical replicates; the average values are presented.

### 2.4 Telomere length analysis

The telomere length was determined using a quantitative PCR (qPCR)-based assay, as described by Joglekar et al. (2020). Genomic DNA was extracted from muscle biopsies of the *MSTN* knockout calf and three age-matched controls using a genomic DNA purification kit (Promega, Cat. No. A1125). Each 10-µL qPCR reaction contained 100 ng genomic DNA, 5 µL SYBR Green master mix, 0.5 µM of each primer (telomere or single-copy reference gene), and nuclease-free water. Telomere-specific primers (TEL-F: 5′-GGT​TGT​TTG​GGT​TTG​GGT​TTG​GGT​TTG​GGT​TTG​GGT​T-3′; TEL-R: 5′-GCT​TGC​CTT​ACC​CTT​ACC​CTT​ACC​CT-3′) and β-globin primers (HBG-F: 5′-GCT​TGT​CAC​AGT​GCA​GCT​TGT​CAT​ACA​GC-3′; HBG-R: 5′-CAC​CAC​CTT​CTT​ACA​GCT​TAC​C-3′) were used. Reactions were performed in triplicate on a CFX96 Real-Time PCR System (Bio-Rad, Hercules, CA, United States) under the following conditions, namely, 95 °C for 3 min, followed by 40 cycles of 95 °C for 15 s and 58 °C for 1 min, and a final melt-curve analysis. The relative telomere length was calculated using the ΔΔCt method, normalizing the telomere repeat amplification to that of the β-globin reference gene, and three technical replicates were performed.

## 3 Results and discussion

**FIGURE 1:**
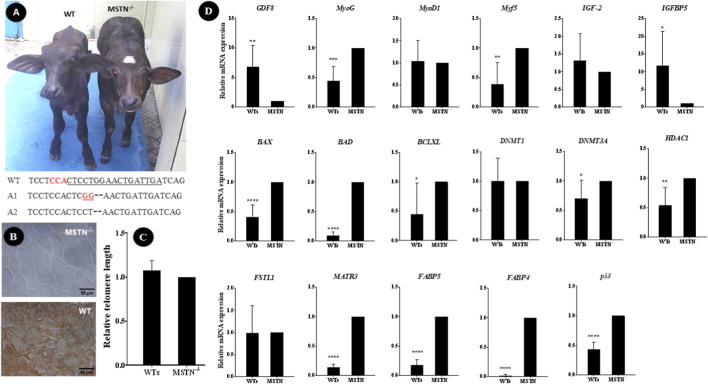
*MSTN* knockout buffalo and the analysis of its telomere length and muscle transcript profiles. **(A)** Representative image of an *MSTN* knockout buffalo (right) and an age-matched control (left), showing the characteristic double-muscle phenotype. The schematic illustrates the mutated *MSTN* alleles generated by CRISPR genome editing. **(B)** Immunohistochemical analysis confirming the loss of MSTN protein expression in the skeletal muscle of knockout buffalo. **(C)** Telomere length is similar between the knockout buffalo and control buffaloes. **(D)** mRNA expression of genes involved in myogenesis, apoptosis, epigenetic regulation, and lipid metabolism in the gluteal muscle of knockout calf and control calves. Three age-matched control buffalo calves’ muscle samples were used for comparative observations.

Genome editing has long been utilized in livestock production to support the development of animals with desirable traits, including improved growth performance and enhanced disease resistance (Yuan et al., 2024). We recently reported the birth of a bi-allelic *MSTN* gene knockout buffalo using CRISPR/Cas9-mediated genome editing with SCNT (Selokar et al., 2025). The calf was born through natural parturition with a birth weight of 32 kg, which was comparable to that of age-matched wild-type calves produced through artificial insemination. The calf exhibited a rapid growth rate and double-muscling phenotype (Figures 1A,B), consistent with previous studies in livestock species (Li et al., 2016; Zhu et al., 2020; Gim et al., 2022). However, after the first 2 weeks of life, it developed progressive respiratory distress, and by approximately 90 days of age, the calf succumbed to severe diarrhea. Despite appropriate care, the condition worsened, and the calf died at 100 days of age. Due to the non-availability of veterinary pathological services at our institute, we were unable to perform a necropsy analysis, which is a major limitation of this study. Thus, the absence of necropsy, pathological, and microbiological examinations restricts our ability to determine the possible cause of death.

As part of routine management in our experimental herd, serum samples are collected from all newborn calves at birth and stored at −80 °C for future analyses. Because the *MSTN* knockout calf died at an early age, we retrospectively examined its stored samples to understand its physiological condition. To understand the status of myogenic regulatory networks, we analyzed the expression of key muscle transcripts (Figure 1D). *MSTN* knockout muscle showed higher mRNA expression of *MyoG* and *Myf5*, while the *MyoD* expression was comparable to that of wild-type controls. These findings align with the established role of *MSTN* as a negative regulator of muscle cell growth through TGF-β/SMAD signaling (Aiello et al., 2018; Dewasi et al., 2025). Apoptosis-related transcripts (*BAD*, *BAX*, *p53*, and *BCLXL*), epigenetic-related transcripts (*DNMT3a* and *HDAC1*), and lipid-related transcripts (*MATR3*, *FABP4*, and *FABP5*) were all highly expressed in *MSTN* knockout muscle, whereas *DNMT1*, *IGF2*, and *FLST1* expression remained unchanged. In contrast, *IGFBP5* expression was reduced in the knockout muscle. These results indicate a balanced myogenic environment that promotes faster muscle growth (Chelh et al., 2009; Sciorati et al., 2016; Massenet et al., 2021).

**FIGURE 2:**
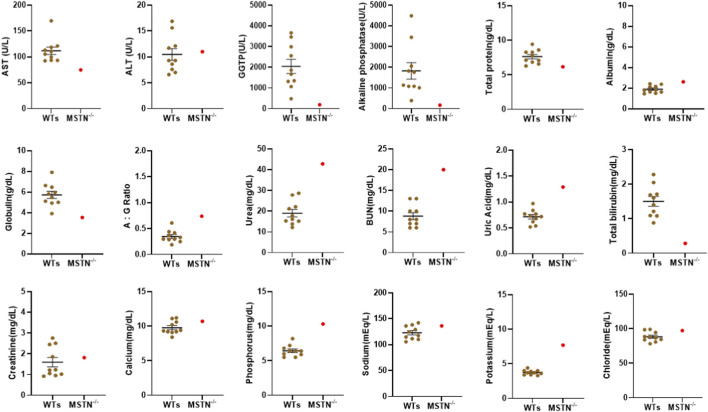
Profile of serum biochemical parameters of the *MSTN* knockout calf and age-matched controls. Ten age-matched control buffalo calves were used for comparative observations. The data point represents a single MSTN knockout animal’s values compared against the mean values of 10 wild-type animals produced through artificial insemination.

**FIGURE 3:**
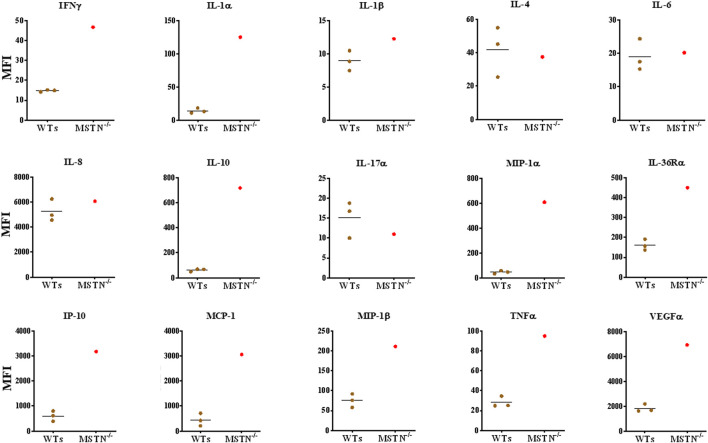
Profile of the circulating cytokines in the *MSTN* knockout calf and age-matched controls. Cytokine levels are expressed as the mean fluorescence intensity (MFI). Three pooled samples from nine age-matched control buffalo calves were used for comparison, with each sample measured in three technical replicates; the average values are presented. The data point represents a single *MSTN* knockout animal’s values compared against the mean values of wild-type animals produced through artificial insemination.

Serum biochemical analysis revealed clear systemic alterations at birth. The levels of GGTP, ALP, total bilirubin, and globulin were markedly lower compared to those in age-matched controls (Figure 2). The reduced globulin concentration, together with an increased albumin-to-globulin ratio, may indicate compromised early immune competence, as neonatal globulin levels are closely linked to passive immunity and disease resistance (Correa et al., 2022; Caliskan et al., 2023). In addition, elevated concentrations of urea, blood urea nitrogen, and uric acid indicate increased protein turnover and altered nitrogen metabolism in the *MSTN* knockout calf. Higher levels of phosphorus and potassium further indicate disturbances in mineral homeostasis and neuromuscular regulation. Serum cytokine profiling also revealed marked immunological changes. Circulating levels of IFN-γ, IL-1α, IL-1β, IL-10, MIP-1α, IL-36Rα, IP-10, MCP-1, MIP-1β, TNF-α, and VEGF-α were elevated in the *MSTN* knockout calf, whereas IL-4, IL-6, and IL-8 remained unchanged, and IL-17α was modestly reduced (Figure 3). These results reveal changes in the cytokine profile characterized by enhanced pro-inflammatory, chemotactic, and angiogenic signaling (Park and Ciofani, 2025). Previous studies have also reported physiological and immunometabolic deviations in *MSTN* gene-edited livestock species (Du et al., 2022; Wen et al., 2022; Gu et al., 2023; Zhao et al., 2025a; Zhao et al., 2025b), supporting the hypothesis that *MSTN* disruption may influence systemic metabolic and immune homeostasis. Due to the limited dataset in this study, further studies are required to elucidate the systemic effects of *MSTN* gene knockout in buffalo.

We also analyzed telomere length, which is an important consideration in genome-edited animals produced using SCNT as reduced telomere length has been linked to developmental abnormalities and early mortality in several species (Burgstaller and Brem, 2017). We found that the telomere length of the *MSTN* knockout calf was comparable to that of age-matched WT buffaloes (Figure 1C), indicating normal telomere aging. Since the findings of this study are derived from a single *MSTN* knockout calf, it is difficult to draw statistical conclusions. Additionally, SCNT is inherently associated with variable developmental outcomes in genome-editing experiments in cattle (Carlson et al., 2016; Laible et al., 2021), and it remains challenging to clearly separate the specific effects of *MSTN* deletion from potential SCNT-related influences. In addition, the absence of an SCNT-derived control group limits our ability to distinguish between the effects of knockout of *MSTN* and those associated with the SCNT procedure. Nonetheless, this case report highlights that deletion of *MSTN* promotes strong muscle hypertrophy in buffalo, and such calves may also exhibit systemic metabolic and immunological alterations with possible health implications. Therefore, intensive veterinary care, including assisted passive immunity transfer, is essential to protect such calves.

## Data Availability

The original contributions presented in the study are included in the article/Supplementary Material; further inquiries can be directed to the corresponding author.
