# Supplementary material for: Clinical outcome of an SCNT-derived MSTN knockout buffalo: a case study
Source: Front Genome Ed. 2026 Jun 9;8:1822169. doi: 10.3389/fgeed.2026.1822169 (PMC13287090; doi:10.3389/fgeed.2026.1822169)
Supplement: Supplementary file 1 [file Table1.docx]

Supplementary Table 1: Primer Sequence

| S.No. | Gene Symbol | Sequence |
| --- | --- | --- |
| 1 | GDF8* | F- ACCTTCCCAGAACCAGGAGAA  R- TCACAATCAAGCCCAAAATCTCT |
| 2 | FABP4* | F- TGGAAACTTGTCTCCAGTGAAA  R- ACCCCCATTCAAACTGATGA |
| 3 | FABP5* | F- TGGGAGAGAAGTTTGAAGAGA  R- TTCCTGATGTTGAACCAATGC |
| 4 | FSTL1* | F- TGCAGACCAGGAGAACAACA  R- GGTTGAGGCACTTGAGGAAC |
| 5 | IGFBP5* | F- GGTTTGCCTGAACGAAAAGA  R- CTTGGGCGAGTAGGTCTCC |
| 6 | MATR3* | F- GGAAAAAAGAACCTTCAGACA  R- CCTCGATCTTGTCCACCTTT |
| 7 | BCL-XL | F- TTGTGGCCTTTTTCTCCTTC  R- GATCCAAGGCTCTAGGTGGT |
| 8 | BAD | F- CCAGAGCATGTTCCAGATCC  R- GTTAGCCAGTGCTTGCTGAG |
| 9 | BAX | F- CCTTTTGCTTCAGGGTTTCA  R- CGCTTCAGACACTCGCTCA |
| 10 | p53 | F- GGAAGAATCACAGGCAGAACTC  R- ACTTCATTCGGACATTCATCCA |
| 11 | HDAC 1 | F-ATCGGTTAGGTTGCTTCAATCTG  R-GTTGTATGGAAGCTCATTAGGGA |
| 12 | DNMT1 | F-CTCAGAAGGGAGATGTGGAG  R-TAGTAGTCACAGTAGCTGAGGA |
| 13 | DNMT3A | F-GTGCTGTCTCTATTCGATGG  R-CCATTCCTGGATATGCTTCTG |
| 14 | MyoD1 | F: CGAACACTATAGCGGCGACT  R: GTAAGTGCGGTCGTAGCAGT |
| 15 | MyoG | F: GCCAGGGCTTATGAAGCAGA  R: CTTTGCCCAGCCAATGAACC |
| 16 | Myf5 | F: CCAAGAATGGTGGTGCCCTA  R: GGGTCCTGGTCTCACTTACC |
| 17 | IGF2 | F: CTCACACACACATTCGGTCG  R: TGGAAGACCCGGGGACAAT |
| 18 | GAPDH | F: CAAGAAGGTGGTGAAGCAGG  R: CCCCAGCATCGAAGGTAGAA |

*These primers are used previously by Lehnert et al., 2007
